# Best Practice for Identification of Classical 21-Hydroxylase Deficiency Should Include 21 Deoxycortisol Analysis with Appropriate Isomeric Steroid Separation

**DOI:** 10.3390/ijns9040058

**Published:** 2023-10-16

**Authors:** Ronda F. Greaves, Monish Kumar, Nazha Mawad, Alberto Francescon, Chris Le, Michele O’Connell, James Chi, James Pitt

**Affiliations:** 1 Victorian Clinical Genetics Services, Murdoch Children’s Research Institute, Parkville, VIC 3052, Australia; monish.kumar@vcgs.org.au (M.K.); nazha.mawad@vcgs.org.au (N.M.); albert.francescon@vcgs.org.au (A.F.); chris.le@vcgs.org.au (C.L.); james.chi@vcgs.org.au (J.C.); james.pitt@vcgs.org.au (J.P.); 2 Department of Paediatrics, University of Melbourne, Parkville, VIC 3052, Australia; michele.oconnell@rch.org.au; 3 Department of Endocrinology, The Royal Children’s Hospital, Parkville, VIC 3052, Australia

**Keywords:** 21-deoxycortisol, method validation, isobaric steroids, interferences, congenital adrenal hyperplasia, liquid chromatography–tandem mass spectrometry, newborn screening, best practice

## Abstract

There are mixed reports on the inclusion and use of 21 deoxycortisol (21DF) as the primary decision marker for classical 21-hydroxylase deficiency. We hypothesize that this may be due to insufficient recognition of the presence and chromatographic separation of isomeric steroids. The aim of this study was to determine the comparative utility of 21DF for screening and diagnosis of CAH due to classical 21-hydroxylase deficiency using a second-tier LC–MS/MS method that included the separation of isomeric steroids to 17OHP and 21DF. For each baby sample, one 3.2 mm dried blood spot was eluted in a methanolic solution containing isotopically matched internal standards. Data were interrogated by univariate and receiver operator characteristic analysis. Steroid profile results were generated for 924 non-CAH baby samples (median gestational age 37 weeks, range 22 to 43 weeks) and 17 babies with 21-hydroxylase deficiency. The ROC curves demonstrated 21DF to have the best sensitivity and specificity for the diagnosis of classical 21-hydroxylase deficiency with an AUC = 1.0. The heatmap showed the very strong correlation (r = 0.83) between 17OHP and 21DF. Our data support 21DF as a robust marker for CAH due to 21-hydroxylase deficiency. We recommend that 21DF be incorporated into routine newborn screening panels as part of the second-tier LC–MS/MS method, follow-up plasma steroid panels, and external quality assurance material.

## 1. Introduction

Newborn screening (NBS) dried blood spot (DBS) steroid analysis for congenital adrenal hyperplasia (CAH) due to classical 21-hydroxylase deficiency is performed by several countries globally [1,2,3,4,5,6,7,8,9,10,11,12,13]. 17-Hydroxyprogesterone (17OHP) has been the principal steroid measured in blood for screening and diagnosis of CAH because in 21-hydroxylase deficiency, it is the immediate precursor (i.e., 17OHP). In addition, a shunt to a normally minor pathway to produce 21-deoxycortisol (21DF) (and its equivalent urine metabolite pregnanetriolone) becomes significant. 21DF is therefore increased in individuals with classical 21-hydroxylase deficiency. This has led to a recent recommendation to replace blood 17OHP with 21DF [14] as the primary marker for 21-hydroxylase deficiency. Contrary to this recommendation, the recent literature contains mixed reports on the value of 21DF with some supporting it as a “Key Screening Marker for 21-Hydroxylase Deficiency” [15] and others, whilst acknowledging the high relevance of 21DF, stating “LC-MS/MS Assay Revealed No Single Marker Differentiates 21OHDs From False-Positive Cases” [16].

**Figure 1:**
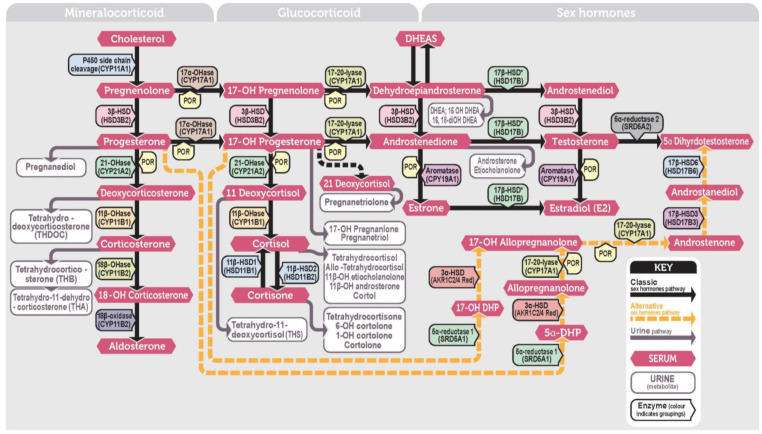
Steroid pathway showing the blood steroids and urine steroid metabolite.; CYP = cytochrome P450, DHP = dihydroprogesterone, HSD = hydroxy steroid dehydrogenase, OH = hydroxy, OHase = hydroxylase, P450 oxidoreductase, POR = cytochrome, SRD = steroid reductase, * after an enzyme name indicates it applies to more than one isoform type. The incidence of CAH varies amongst the six enzyme defects with 21-hydroxylase (caused by a deletion or mutation in the CYP21A2 gene) and then 11β-hydroxylase (CYP11B1) deficiencies the most common (1:15,000 and 1:100,000, respectively) with both resulting in increased 17OHP levels in the blood. 17α-Hydroxylase (CYP17A1), 3β-hydroxysteroid dehydrogenase (HSD3B2), and lipoid adrenal hyperplasia (STAR), are all rarer forms that arise due to deficiencies of enzymes earlier in the biosynthetic pathway and would result in low levels of 17OHP. An additional form of CAH related to deficiency of the P450 oxidoreductase enzyme, which is a coenzyme for 21-hydroxylase, 17α-hydroxylase and 17,20-lyase is also recognized and results in a mixed picture of increased 17OHP but low androgens, e.g., androstenedione [23,24]. Newborn screening pathways are focused on detecting classical CAH due to 21-hydroxylase deficiency. Figure reproduced with permission from [25]. Copyright 2018 Elsevier Ltd.

Prior to the advent of liquid chromatography—tandem mass spectrometry (LC–MS/MS), definitive biochemical phenotypic classification of CAH was usually conducted by urine steroid metabolome analysis with gas chromatography–mass spectrometry (GC–MS) [17]. In this method, the two most common forms of CAH, classical 21-hydroxylase and 11 beta-hydroxylase deficiency could easily be distinguished by the urine steroid metabolites pregnanetriolone and tetrahydro-11-deoxycortisol, respectively [18,19]. In particular, urine pregnanetriolone has been considered the definitive steroid for the diagnosis of classical 21-hydroxylase deficiency in both preterm and full-term babies for decades [20]. This orthogonal process supports the supposition that 21DF is the key marker. Figure 1 shows the steroid pathway and relationship between blood and urine steroid metabolites.

The underlying question then is, why do some laboratories not support the proposition of 21DF as a key marker? We hypothesize that this may be due to insufficient recognition of the presence of isomeric steroids and, therefore, not taking appropriate attention to the separation of these chromatographically. This supposition is supported by a recent global survey of laboratories performing LC–MS/MS 17OHP analysis, where approximately half did not know or account for common steroid isomers [21] and the demonstration of clinically relevant isomers to 17OHP [22]. Equivalent work is not available for 21DF.

As part of the Victorian newborn screening implementation strategy for CAH, we developed a robust and specific second-tier LC–MS/MS method that separated the isomeric steroids for 17OHP and 21DF. Using this method, we aimed to determine the comparative utility of 21DF for screening and diagnosis of CAH due to classical 21-hydroxylase deficiency.

## 2. Materials and Methods

### 2.1. Victorian NBS Program Samples

The NBS program for the State of Victoria, Australia is located within the Royal Children’s Hospital Melbourne campus at the Victorian Clinical Genetics Services (VCGS). The NBS laboratory services around 90 maternity service providers, testing approximately 80,000 newborns annually, including for CAH since 2022 [26]. The dried bloodspot (DBS) sample is usually collected via a capillary heel prick onto Whatman 903 paper when the baby is 36 to 72 h of age [27,28]. On arrival to the laboratory, one 3.2 mm spot is punched from the card for the first tier 17OHP immunoassay which is analysed using the Perkin–Elmer (Turku, Finland) GSP^®^ Neonatal 17OHP kit. For results ≥20.0 nmol/L blood (approximating the 99th centile), another 3.2 mm punch is taken for second-tier LC–MS/MS steroid analysis.

### 2.2. Patients’ Samples

Over a seven-month period in 2022, 46,056 routine NBS DBS samples were analyzed for 17OHP by this immunoassay method. In addition, as part of the initial method validation, archival DBS samples were retrieved from clinically diagnosed CAH patients for analysis. These samples had been stored from six months to ten years (median three years) under ambient conditions. These samples were used to set the cut-offs and these limits were applied to routine screening for CAH in our jurisdiction. In addition, prospective screen positive patients presenting after this cohort study period were used to review the robustness of these cut-offs.

### 2.3. Mass Spectrometry Materials

Chemicals: Methanol Optima LC–MS grade (FSBA456-4) and Formic acid Optima LC–MS grade (A117-50) were purchased from Thermo Fisher Scientific (Scoresby, VIC, Australia). 17OHP was purchased from the National Measurement Institute of Australia (NSW, Australia). All other quantitated steroids (11-deoxycortisol, 21-deoxycortisol, androstenedione, cortisol, cortisone, progesterone and testosterone), isomeric steroids (16-hydroxyprogesterone, 11-deoxycorticosterone, 11-deoxycorticosterone and corticosterone) and internal standards (17OHP [13C3], Androstenedione [13C3], Cortisol [13C3], Cortisone [13C3], Progesterone [13C3], 11-deoxycortisol [D5], 21-deoxycortisol [D8] and Testosterone [D3]) were purchased from PM Separations, (Capalaba, QLD, Australia) (Appendix A—Table A1). Blank whole blood was purchased from UTAK (Valencia, CA, USA) via their Australian distributor PM Separations.

Calibrator preparation: All steroids, except for 17OHP, were purchased in concentrated stock form in methanol. 17OHP powder was weighed out and reconstituted in methanol to prepare a standard stock solution. The steroid stocks were diluted using methanol and spiked into UTAK blood. This produced the highest calibrator. The other calibrator levels were produced by mixing the spiked blood used initially for the highest calibrator with blank UTAK base material in different ratios. The concentration range for the linearly related calibration set were: 11-deoxycortisol 0–99 nmol/L, 17OHP 0–204 nmol/L, 21DF 0–101 nmol/L, androstenedione 0–923 nmol/L, cortisol 15–925 nmol/L, cortisone 13–928 nmol/L, progesterone 0 to 99 nmol/L and testosterone 3–102 nmol/L. An amount of 50 uL of calibrator was spotted on the filter paper (Whatman 903) and air-dried overnight in ambient condition. The prepared calibrators were stored at −80 °C.

Commercial internal quality control material at two levels was purchased from Australian Scientific Enterprise (ASE, Hornsby, NSW, Australia). Our laboratory routinely participates in the Centres for Disease Control NBS Program and the Royal College of Pathologists of Australasia Quality Assurance Program (RCPAQAP, St Leonards NSW, Australia) dried blood spot program samples with acceptable performance in both. The method and laboratory received accreditation to ISO15189:2012 in April 2022 and was subsequently registered as an in-house IVD with the Therapeutic Goods Administration of Australia.

### 2.4. Second-Tier Analytical Method

A single 3.2 mm DBS sample per card was punched and eluted on a 96-well plate using 220 µL of 95% methanol containing the internal standards for 45 min in a thermomixer at 37 °C. An amount of 150 µL of the supernatant was transferred to a new plate and air-dried at 65 °C. The samples were reconstituted with 200 µL of 40% methanol in water before analysis.

The Waters Acquity UPLC I class system (Waters Corporation; Milford, MA, USA) was used for steroid separation by a Waters CSH C18 column (2.1 mm × 50 mm,1.7 µm). The steroids and their corresponding internal standards were eluted by a 9.6 min gradient, starting with 40% solvent A (0.1% *v*/*v* formic acid in 2% methanol) to 60% solvent B (0.1% *v*/*v* formic acid in methanol). Steroids were separated chromatographically to ensure distinct elution of clinically relevant isobaric steroids to 17OHP and 21DF [22]. Clinically relevant isobaric steroids for 17OHP were 11OHP, 16OHP and 21OHP; for 21DF they were 11-deoxycortisol and corticosterone. The mass selective detection was performed on a Waters Xevo TQXS mass spectrometer in positive electrospray ionization (ESI) mode with multiple reactions monitoring (MRM) utilized for quantitation and qualification of each steroid (Table 1 and Appendix A—Figure A1).

Steroid quantitation was performed by the TargetLynx Manager in the Waters MassLynx V4.2 software by linear regression of peak area ratios of steroids/labelled internal standards against the calibrator concentrations with 1/x weighting. Results calculated from the qualification MRMs must differ from the quantitation MRM results by <20%.

### 2.5. Method Validation Studies and Acceptance Criteria

The formal method validation study included assessment of imprecision, bias, linearity, method comparison, external quality program performance and assessment of isobaric steroids to 17OHP and 21DF (Appendix A including Table A2).

- Precision—three experiments were performed to determine imprecision: (a) overall method within run imprecision using n = 20 replicates; (b) LC–MS/MS within run injection replicate imprecision using one vial injected n = 20 times; and (c) between run imprecision assessed with the bi-level IQC material. Acceptance criteria was set to be 50% of the biological variation, i.e., this represents the desirable imprecision for each analyte and ideally the CV should be <10%.
- Linearity—initial linearity was assessed with the calibration curve and then using the same sample matrix using a mixture of high (IQC) and low (pooled normal patient) and using non-matrix matched with high (standard) and low (pooled normal patient). Acceptance criteria were set as follows: Linearity—the calibration slope should be r = 0.99. Linearity outside the upper limit requires formal linearity studies to determine the upper reportable range. The Linchecker version 1.1.2.0 by Philippe Marquis (Metz, France) was used to assess this and if “linearity” is determined, then it is acceptable.
- Bias—assessed through recovery study and the external quality assurance sample comparison study. Note: Some, but not all steroid standards, are traceable to high order reference materials or methods listed in the JCTLM database. Acceptance criteria were set as follows: Bias–recovery studies with results between 85 and115% were acceptable. RCPAQAP and CDC EQA were the main determinants of actual bias with acceptable EQA performance.
- Method comparison—EQA samples and immunoassay GSP 17OHP results. Acceptance criteria were set. Method comparison—95% CI for Passing Bablok slope to include 1, 95% CI of Bland Altman plot to include zero. For analytes where the CI decision is not met, an evaluation of the clinical significance of deviation is made to determine acceptance. This is, however, limited as this is a new method and not a change. EQA for all steroids and first- and second-tier 17OHP will be compared but noting they are different measurands.
- Uncertainty of measurement—this was based on a coverage factor of 2 for between-run imprecision of IQC.
- Limit of blank/detection/quantification—assessed low (pooled normal patient) at different dilutions with (n = 10) of imprecision (standard deviation) for each calculation. Acceptance criteria were set as follows: Limit of quantitation (LOQ) of 20% or S/N of 10 and limit of detection (LOD) with a S/N of 2, which is relevant for analytes that are usually not present in patient samples, e.g., 21 DF. The LOQ should meet clinical relevance and fall at least into or below the cut-offs (analyte-specific).
- Carry-over—assessed by quantification of a blank injection after a high concentration sample (standard LV 5). Carry-over—less than 2%.
- Interference—known isomeric steroids to 17OHP (11-alpha and 11-beta hydroxy progesterone, 16-hydroxyprogesterone, and 21-hydroxyprogesterone) and 21DF (11-deoxycortisol and corticosterone) were tested to ensure resolution from their respective measurands. Acceptance criteria were set as follows: Interference—0% bias.
- Decision limits—cut-offs were assessed by running (1) normal neonates, (2) retrieval of known patients’ DBS samples, and (3) comparison with Australasian cut-offs already in place. Decision limits were determined in-house by visual assessment, centiles, and receiver operator sensitivity and specificity characteristics.
- Assessment of fitness for clinical purpose—the overall decision to implement the quantitation of each steroid was based on the method validation results and the overall performance against clinical need. Fitness for purpose—reviewed against the clinical utility of each analyte.

### 2.6. Statistical Analysis

The patient data were interrogated using Stata-18.0 and MetaboAnalyst-5.0. Investigations included the spread of the data, graphical presentation of outliers and concentration, ratio and heatmap of Peason correlations, ROC analysis and quantile regression. Microsoft Excel and the Philippe Marquis (Metz France) Method Validation (version 1.19), Linearity (version 1.1.2.0) and QC (version: stand-alone 3.21) software were used for the method validation studies.

## 3. Results

### 3.1. Method Validation and Steroid Isomer Separation

**Figure 2:**
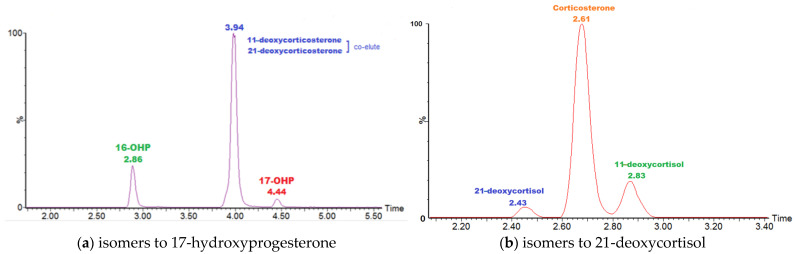
LC–MS/MS chromatograms of methanolic solution of potentially interfering isobaric steroids illustrating separation: Isomeric separation from (**a**) 17OHP and (**b**) 21DF. Note 1: 11-deoxycorticosterone is also known as deoxycorticosterone and 21-hydroxyprogesterone. Note 2: 21-deoxycorticosterone is also known as 11-hydroxyprogesterone.

**Figure A1:**
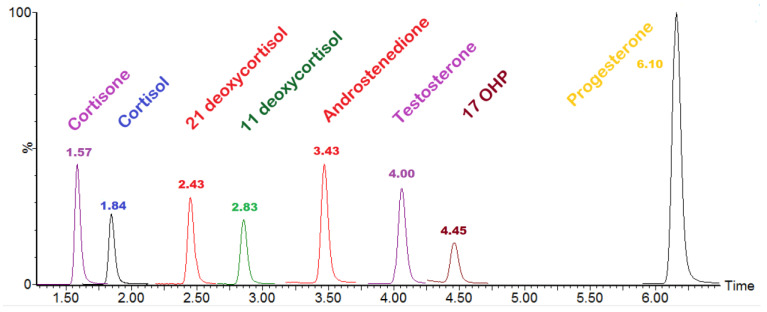
LC–MS/MS chromatogram of the eight-steroid panel used for the second-tier CAH profiling. Panel is of the pure steroids in methanol.

The method was successfully validated and deemed fit for clinical purpose. The results and outcomes are presented in Appendix A Table A2, and an associated chromatogram showing the eight steroids is presented in Appendix A Figure A1. The dried blood spot LC–MS/MS steroid method successfully separates the known isomeric interferences to 17OHP and 21DF (Figure 2).

**Figure A2:**
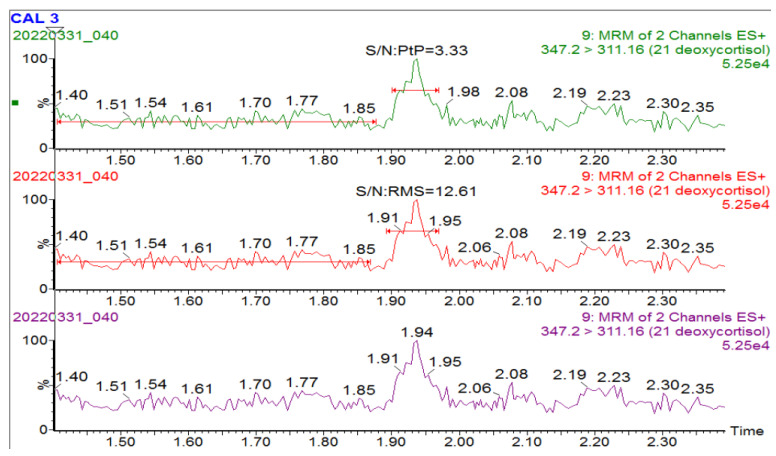
Baseline response for trace 347.2 > 311.2 of Cal 0 for limit of detection of 21DF.

21DF is not normally detected in patient samples using current methods. There is continued discussion on how to treat and report low results associated with analytes that should not be routinely detected in non-disease/treated patients. We have taken the approach of using detection limit rather than quantification limit for 21DF. This is important for low (but abnormal) results identified. In this approach, whilst we quantify the data for reporting, it is truly a qualitative value at low but detectable levels. Appendix A Table A3 and Appendix A Figure A2 show the results of the experiments performed for 21DF limit of detection.

### 3.2. Statistical Analysis of Steroid Data

Using this method for the second-tier analysis (from the 46,056 DBS samples analysed by GSP immunoassay over a seven-month period between April and November 2022), 924 DBS samples were analysed. This included:

- 35% (n = 322) of non-CAH babies that were full term.
- 28% (n = 260) of non-CAH babies that were late preterm, i.e., 32 to <37 weeks.
- 34% (n = 313) of non-CAH babies had a recorded gestational age of <32 weeks.
- 3% (n = 29) of non-CAH babies did not have a gestational age recorded and were excluded from analysis if there was a division between preterm and full-term birth.
- Two female babies were identified prospectively to have CAH due to 21-hydroxylase deficiency within this data analysis period (both were full-term neonates).
- A further 14 archival DBS samples were retrieved from children with known 21-hydroxylase deficiency (diagnosed prior to initiation of CAH testing in the NBS program).
- One additional archival card was retrieved as part of the diagnostic work up of a child who presented at eight months with virilization and confirmed by our method to have 21OHase deficiency.

Of the 17 babies in total with CAH due to 21Ohase deficiency, two had equivocal results using the ratio and 17OHP but were identified with 21DF because it was such an outlier.

**Figure 3:**
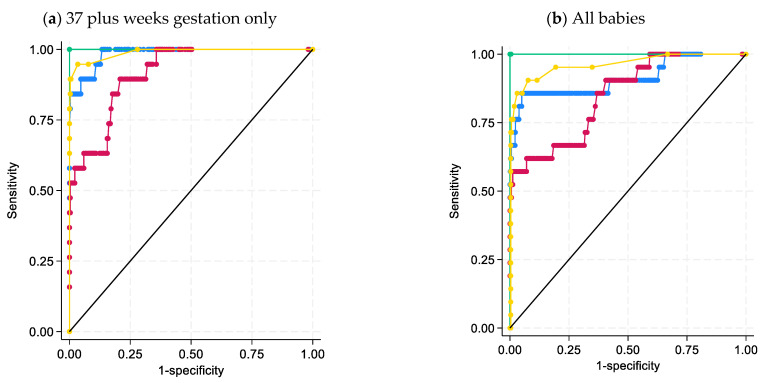

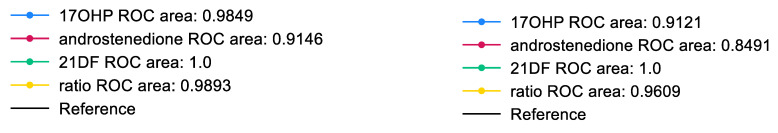
ROC compare analysis using 17OHP, androstenedione, 21DF and the ratio ((17OHP+androstenedione)/cortisol) by mass spectrometry (**a**) for babies 37 plus weeks gestation, n = 322 non-CAH and n = 16 CAH; and (**b**) all babies n = 924 non-CAH and n = 17 babies with diagnosed 21OHase deficiency.

**Figure 4:**
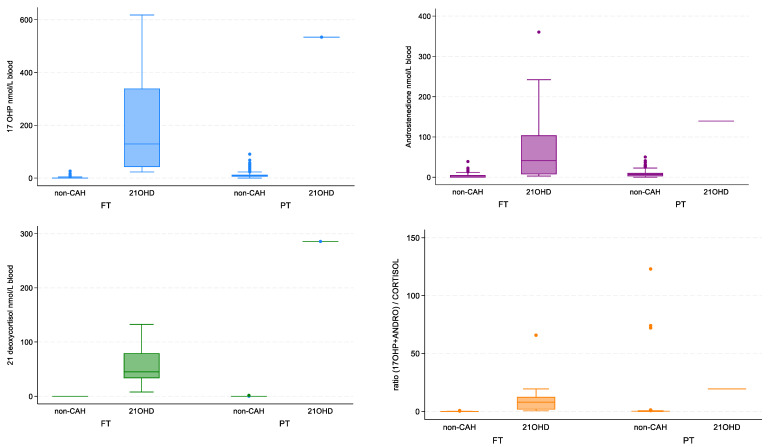
Box and whisker plots comparing DBS results from non-CAH babies (full-term (FT) n = 322 preterm (PT) n = 573, and n = 29 excluded as no gestational age) with diagnosed 21OHase deficiency (21OHD) dried blood spot samples (n = 16 FT and n = 1 PT which was for a 36 weeks’ gestation baby). FT—full-term babies 37 or more weeks gestation); and PT—preterm babies 36 or less weeks gestation.

**Figure 5:**
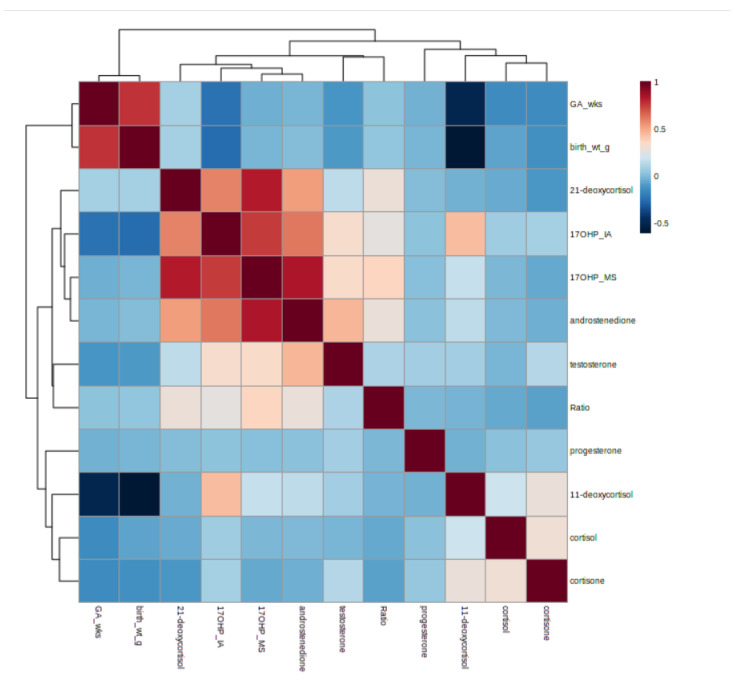
Correlation heatmap demonstrating the relationship between the steroids measured in the second-tier LC–MS/MS panel. Ratio is (17OHP+androstenedione)/cortisol. For 17OHP the IA designated immunoassay and MS for mass spectrometry

The ROC curves generated from this data set demonstrated 21DF to have the best sensitivity and specificity for the diagnosis of 21-hydroxylase deficiency with an AUC = 1.0 (Figure 3). This is also shown in the box and whisker plots, with 21DF giving the best discrimination between non-CAH and 21-hydroxylase deficiency (Figure 4). The heatmap also supports the inclusion of 21DF with a correlation ofr = 0.83 between 17OHP and 21DF (Figure 5).

Conservative cut-offs were established for each steroid based on visual inspection, centiles and final confirmation with the receiver operator sensitivity and specificity characteristics (Figure 3). Mass spectrometry steroid cut-offs were developed in the initial pilot and remained unchanged during the study period: 17OHP (cut-off < 20 nmol/L blood had an associated sensitivity of 78% and specificity of 98%); androstenedione (cut-off < 20 nmol/L blood had an associated sensitivity of 78% and specificity of 97%); 21DF (cut-off < 1.0 nmol/L blood had an associated sensitivity of 100% and specificity of 99%); cortisol (20–800 nmol/L blood with some extreme high values seen in premature infants); and ratio of 17OHP+androstenedione/cortisol (cut-off < 1 nmol/nmol had an associated sensitivity of 89% and specificity of 99%). Only 21DF had a cutoff point where there was both 100% sensitivity and specificity, which was at 7.9 nmol/L blood. Results outside of these limits were sent for higher level review (authors RG and/or JP) and final interpretation considering the overall profile and the baby’s gestation.

### 3.3. Classical CAH Due to 21-Hydroxylase Deficiency-Patient Review

Reviewing archived DBS data from the 15 babies with known CAH, two patients had mildly increased 17OHP on first-tier testing and mildly increased 17OHP and normal ratio ((17OHP+androstenedione)/cortisol) on second-tier testing. These screening results were considered equivocal. However, 21DF was significantly increased in both providing definitive confirmation of the diagnosis.

After the seven-month data gathering period was completed, two additional babies were identified as part of the NBS program. Therefore in total in the first year of screening four classical CAH babies (two female and two male) were identified, giving an initial incidence of approximately 1:20,000. One of these screen positive cases in particular exemplifies the importance of 21DF in the decision pathway; Table 2.

Additional information related to the results section can be found in the Appendix A.

## 4. Discussion

The current work presents data demonstrating that 21DF is the superior marker to identify CAH due to classical 21-hydroxylase deficiency. Importantly, as part of our Victorian NBS implementation strategy, we have chromatographically separated isomeric steroids for 21DF and 17OHP, and with this robust method in place, 21DF demonstrated an ROC curve of 1.0; thus, it is our primary decision marker for referral for clinical follow-up. 17OHP and the ratio of 17OHP + androstenedione/cortisol continue to remain important measurands to give an overall steroid pattern.

The CAH group of adrenal biosynthetic enzyme deficiencies can result in atypical genital appearance (+/− difficulty with sex assignment), early salt wasting and adrenal crises, as well as unexplained sudden death in infancy which can be avoided with timely diagnosis [29]. Newborn screening for the timely detection of classical CAH due to 21-hydroxylase deficiency has been conducted in many jurisdictions for decades with the aim of preventing the clinical consequences. However, as screening is aimed at doing more good than harm, the debate surrounding the number of false positive 17OHP immunoassay results (especially in preterm neonates) has slowed the update of this screening condition. Fortunately, with the improvements in LC–MS/MS analytical sensitivity, a second-tier multiplex steroid profile significantly reduces the false positive screening [2].

Both 21DF and 17OHP have clinically relevant isobaric steroids that will cause irregular errors in quantification if not chromatographically separated. In a global survey of laboratories measuring 17OHP by LC–MS/MS, a significant number (45%) had not checked for interferences from clinically relevant isobaric steroids [21]. Clinically relevant isobaric steroids for 17OHP have been identified as 11-hydroxyprogesterone (also known as 21-deoxycorticosterone), 16-hydroxyprogesterone and 21-hydroxyprogesterone (also known as 11-deoxycorticosterone or deoxycorticosterone), sharing a molecular weight of 330.46 g/mol [22]. Likewise, for 21DF, 11-deoxycortisol and corticosterone share a common molecular weight of 346.46 g/mol. A failure to separate these isobaric steroids will cause inaccurate results and the recent global survey highlights that laboratories do not always consider or test for isobaric interferences [21]. We, therefore, postulate that this is a likely cause of the continued debate.

Antenatal administration of glucocorticoids, which is commonly undertaken in mothers of preterm babies, may suppress the steroid pathway. A single dose of antenatal steroids, e.g., Betamethasone, prior to blood collection is not thought to significantly alter the steroids [30]. However, infants treated with multiple antenatal courses of steroids had lower blood 17OHP and thus the conclusion was their potential to have false negative results for CAH [31,32]. A limitation of our study was not knowing the glucocorticoid status of the preterm babies analysed for second-tier steroids. This and persistence of the foetal adrenal gland, warrant the recollection of DBS on preterm infants [32].

Ideally, irrespective of the jurisdiction the baby is born in, we want the same decision cascade for CAH. Our findings for the inclusion of 21DF are consistent with reports based on blood LC–MS/MS analysis and the urine metabolite [14,20,33,34]. For the inclusion of 21DF, the analytical steroid methods need to be sufficiently robust to ensure appropriate separation of steroids isobaric to 21DF (and 17OHP) to avoid irregular errors and false positives [22]. The inability to chromatographically resolve interfering isobaric steroids may lead to poor analytical specificity for 21DF with consequent apparently poor screening performance. Considering the evidence for inclusion of 21DF presented here, we recommend that each jurisdiction harmonise their second-tier steroid assay by incorporating 21DF, with appropriate isobaric separation, into their LC–MS/MS steroid panel.

## 5. Conclusions

Our data support 21DF as a robust marker for CAH due to 21-hydroxylase deficiency. To achieve accuracy, this analyte requires chromatographic separation from isobaric steroids. We recommend that 21DF be incorporated into routine NBS panels, follow-up plasma steroid panels, and external quality assurance material.

## Figures and Tables

**Table 1:** Steroid and internal standard MS File parameters. Analytical targets are shown in grey, internal standards in blue and steroids studied qualitatively are in white. * Steroids isobaric to 17OHP; ** Steroids isobaric to 21DF. In our neonatal population we have identified an additional peak isobaric to 21DF.

Group	Steroid	MW (g/mol)	Parent Ion	Quantifier		Qualifier		Cone Voltage	RT
				Ion	CE	Ion	CE		
1	Cortisol	362.47	363.1	121.0	20	327.2	14	20	1.83
	21-Deoxycortisol	346.46	347.2	311.2	16	121.0	32	25	2.43
	11-Deoxycortisol **	346.46	347.2	97.1	25	109.0	25	20	2.83
	Androstenedione	286.41	287.2	97.1	20	109.0	20	20	3.43
	17OHP	330.46	331.3	97.1	20	109.2	26	20	4.44
	Cortisone	360.45	361.2	163.1	22	121.1	30	20	1.57
	Testosterone	288.42	289.2	97.1	20	109.0	24	30	4
	Progesterone	314.47	315.2	97.1	20	109.0	22	25	6.1
2	16-OHP *	330.46	331.2	97.0	22	109.0	24	20	2.86
	11-Deoxycorticosterone *	330.46	331.2	97.1	24	109.0	32	20	3.9
	21-Deoxycorticosterone *	330.46	331.2	295.2	14	121.1	16	20	3.95
	Corticosterone **	346.46	347.2	120.7	20	293.5	15	25	2.50
	Unidentified steroid **	346.46	347.2	311.2	16	121.0	32	25	2.35
3	Cortisol [13C3]	365.44	366.2	124.0	20			20	1.83
	21-Deoxycortisol [D8]	354.51	355.0	319.0	16			25	2.43
	11-Deoxycortisol [2H5]	351.49	352.2	100.0	25			20	2.83
	Androstenedione [13C3]	289.39	290.2	100.0	20			20	3.43
	17OHP [13C3]	333.44	334.2	100.0	20			20	4.44
	Cortisone [13C3]	363.42	364.2	166.1	22			20	1.57
	Testosterone [D3]	291.44	292.2	97.1	20			30	4
	Progesterone [13C3]	317.44	318.1	100.0	20			25	6.1

**Table 2:** Results from one male baby prospectively screen positive who was subsequently confirmed to have CAH due to 21-hydroxylase deficiency. The baby was referred based on the initial result. The additional results show the subsequent increase in steroids.

Parameter	First Sample	Second Sample	Third Sample	Cut-Off
Patient’s age at sample collection (days)	2 days (50 h of age)	5 days	6 days	
17OHP–IA (nmol/L)	52	122	125	1–20
17OHP–MS (nmol/L)	16	68	100	<20
Androstenedione (Δ4A) (nmol/L)	10	11	18	<20
21-deoxycortisol (21DF) (nmol/L)	10	7	8	<1
11-deoxycortisol (S) (nmol/L)	1	4	6.8	<10
Cortisol (F) (nmol/L)	47	60	53	20–800
Ratio (17OHP + Δ4A)/F	0.6	1.3	2.2	<1

## Data Availability

Please email the corresponding author if you would like to discuss the background data further.

## Appendix A

Additional Information Related to the Steroid Method and its Validation.

**Table A1:** Steroids used to prepare calibrators and their related internal standards.

Consumable	Catalogue Number
Steroids Standards—PM Separations and NMIA	
17OHP-NMI	H5752
11-Deoxycortisol 1 mg/mL acetonitrile 1 mL	COR-1627-1LA LIP
21-Desoxycortisol 100 ug/mL 1 mL	S13407-0.1 ISO
Androstenedione 1 mg/mL methanol 1 mL	AND-1489-1LM LIP
Cortisol 1 mg/mL methanol 1 mL	COR-1630-1LM LIP
Cortisone 1 mg/mL methanol 1 mL	COR-1633-1LM LIP
Progesterone 1 mg/mL acetonitrile 1 mL	PRO-1683-1LA LIP
Testosterone 1 mg/mL methanol 1 mL	TES-842-1LM LIP
Internal Standard—PM Separations	
17a-Hydroxyprogesterone-[2,3,4-13C3] 100 ug/mL 1 mL	S10333-0.1 ISO
Androst-4-ene-3,17-dione-[13C3] 100 ug/mL 1 mL	S9044-0.1 ISO
21-Deoxycortisol-D8 (2,2,4,6,6,21,21,21-D8) 100 ug/mL 1 mL	D-076-1ML CER
11-Deoxycortisol-[D5] 100 ug/mL 1 mL	S9012-0.1 ISO
Cortisol-[13C3] 100 ug/mL 1mL	S14465-0.1 ISO
Cortisone-[13C3] 100 ug/mL 1mL	S14466-0.1 ISO
Testosterone-D3 1 mg/mL methanol 1 mL	TES-1149-1LM LIP
Progesterone-[2,3,4-13C3] 100 ug/mL 1 mL	S10314-0.1 ISO
Internal Quality Controls—Australian Scientific Enterprise and Inhouse	
ASE level 2 and 5	920.2.03 & 920.5.03
Mobile Phases—Thermofisher Scientific	
Methanol Optima LC-MS grade	FSBA456-4
Formic acid Optima LC-MS grade	A117-50

**Table A2:** Method validation summary.

Analyte	Recovery (%)	Linearity (R^2^)	Sample Prep Imprecision (%)	Injections Imprecision (%)	Between Run Imprecision ASE LV 2 (CV %)	Between Run Imprecision ASE LV 5 (CV %)	Carryover (%)	Interference	S/N: Peak to Peak	Uncertainty of Measurement (%)	Cut-Off (nmol/L)
17 OHP	100.7	0.99	8.4	2.0	7.1	6.8	0	Not detected	5.46	16.8	<20
11-Deoxycortisol	102.7	0.99	8.9	2.9	7.3	7.5	0	Not detected	31.91	17.8	<10
21-Deoxycortisol	100.9	0.98	12.5	6.2	8.3	9.7	0	Not detected	45.16	25.0	<1 *
Androstenedione	101.9	0.99	6.5	0.8	7.2	9.6	0	Not detected	3.49	12.9	<20
Cortisol	104.1	0.99	9.3	2.4	6.8	6.9	0	Not detected	3.33	18.6	20–800
Cortisone	103.5	0.99	6.6	1.3	N/A	N/A	0	Not detected		13.2	40–400
Testosterone	105.6	0.99	10.2	2.6	N/A	N/A	0	Not detected		20.3	<5
Progesterone	104.2	0.99	8.1	1.8	N/A	N/A	0	Not detected		16.1	<50

* for 21DF, results above 1 nmol/L are sent for higher level review that includes checking of the steroid pattern and the chromatograms.

**Table A3:** Limit of blank of Utak blood (spotted onto DBS) run 10 times for 21DF. Signal-to-noise estimates.

Name	Material		Value
Blank 1	Cal 0		1.627 × 10^4^
Blank 2	Cal 0		1.678 × 10^4^
Blank 3	Cal 0		1.714 × 10^4^
Blank 4	Cal 0		1.634 × 10^4^
Blank 5	Cal 0		1.597 × 10^4^
Blank 6	Cal 0		1.777 × 10^4^
Blank 7	Cal 0		1.678 × 10^4^
Blank 8	Cal 0		1.705 × 10^4^
Blank 9	Cal 0		1.653 × 10^4^
Blank 10	Cal 0		1.724 × 10^4^
**Signal-to-Noise Ratio**			
**Steroids**	**Conc (nmol/L) used to determine S/N**	**S/N: RMS**	**S/N: Peak to peak**
17OHP	10.25	25.13	5.46
Androstenedione	46.17	139.9	31.91
Cortisol	45.5 + endogenous	214.72	45.16
11 deoxycortisol	4.95	17.19	3.49
21 deoxycortisol	20.28	12.61	3.33
